# Genes Involved in the Endoplasmic Reticulum *N*-Glycosylation Pathway of the Red Microalga *Porphyridium* sp.: A Bioinformatic Study

**DOI:** 10.3390/ijms15022305

**Published:** 2014-02-07

**Authors:** Oshrat Levy-Ontman, Merav Fisher, Yoram Shotland, Yacob Weinstein, Yoram Tekoah, Shoshana Malis Arad

**Affiliations:** 1 Department of Biotechnology, Rager Ave., Ben-Gurion University of the Negev, Beer-Sheva 8410501, Israel; E-Mails: meravfish@gmail.com (M.F.); yoram.tekoah@protalix.com (Y.T.); arad@bgu.ac.il (S.M.A.); 2 Department of Chemical Engineering, Sami Shamoon College of Engineering, Basel/Bialik sts., Beer-Sheva 8410001, Israel; E-Mail: yshotlan@sce.ac.il; 3 Department of Microbiology and Immunology, Rager Ave., Ben-Gurion University of the Negev, Beer-Sheva 8410501, Israel; E-Mail: yacob@bgu.ac.il

**Keywords:** bioinformatics, contigs, microalgae, *N*-glycosylation, *Porphyridium* sp., red algae

## Abstract

*N*-glycosylation is one of the most important post-translational modifications that influence protein polymorphism, including protein structures and their functions. Although this important biological process has been extensively studied in mammals, only limited knowledge exists regarding glycosylation in algae. The current research is focused on the red microalga *Porphyridium* sp., which is a potentially valuable source for various applications, such as skin therapy, food, and pharmaceuticals. The enzymes involved in the biosynthesis and processing of *N*-glycans remain undefined in this species, and the mechanism(s) of their genetic regulation is completely unknown. In this study, we describe our pioneering attempt to understand the endoplasmic reticulum *N*-Glycosylation pathway in *Porphyridium* sp., using a bioinformatic approach. Homology searches, based on sequence similarities with genes encoding proteins involved in the ER *N*-glycosylation pathway (including their conserved parts) were conducted using the TBLASTN function on the algae DNA scaffold contigs database. This approach led to the identification of 24 encoded-genes implicated with the ER *N*-glycosylation pathway in *Porphyridium* sp. Homologs were found for almost all known *N*-glycosylation protein sequences in the ER pathway of *Porphyridium* sp.; thus, suggesting that the ER-pathway is conserved; as it is in other organisms (animals, plants, yeasts, *etc*.).

## 1. Introduction

Glycosylation is one of the most fundamental post-translational protein modifications in eukaryotes. The sugars that are added to the protein during this process affect the physicochemical properties and polymorphism of proteins- e.g., their stabilization, protection, targeting, and direct activity [1–4].

**Figure 1.**
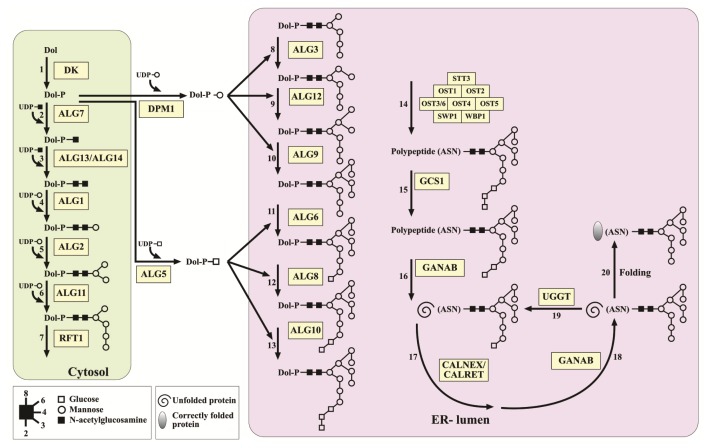
Schematic representation of the pathway for *N*-linked glycoprotein biosynthesis. In this process, a Man_5_GlcNAc_2_-PP-dolichol lipid-linked precursor intermediate is assembled. This intermediate is then extended in the lumen of the ER until Glc_3_Man_5_GlcNAc_2_-PP-dolichol lipid-linked precursor is completed. The Glc_3_Man_5_GlcNAc_2_ oligosaccharide is then transferred onto the target nascent protein to form a protein precursor. This protein precursor is then deglucosylated/reglycosylated to ensure quality control of the neosynthetized protein. The proteins involved are listed in the yellow squares and annotated in Table S1.

Protein *N*-glycosylation in eukaryotes takes place along the endoplasmic reticulum (ER)-Golgi pathway, beginning with the production of a precursor. In this process, a Man_5_GlcNAc_2_ core-oligosaccharide attached to the lipid carrier dolichyl pyrophosphate (Man_5_GlcNA_c2_-PP-dolichol lipid-linked precursor intermediate) is assembled by the stepwise addition of monosaccharides to dolichol pyrophosphate on the cytosolic side of the ER [5] (stages 1–7, Figure 1). This intermediate precursor is then extended in the lumen of the ER until a Glc_3_Man_9_GlcNAc_2_-PP-dolichol lipid-linked precursor is completed [5] (stages 8–13, Figure 1). Later, the oligosaccharide Glc_3_Man_9_GlcNAc_2_ is transferred from the dolichol phosphate to the growing, nascent polypeptide chain via the nitrogen atom of an asparagine amino acid residue by Dolichyldiphosphoryloligosaccharide-protein, or oligosaccharyltransferase (OST) in the lumen of the rough ER (stage 14, Figure 1) [6,7]. The asparagine must be part of the consensus sequence, Asparagine-X-Serine/Threonine (where X is any amino acid except Proline) [8]. Following the oligosaccharide transfer, membrane bound Mannosyl-oligosaccharide glucosidase I (GCS1) [9] and the soluble Alpha 1,3-glucosidase II (GANAB), which is composed of two subunits α and β [10], remove the α1,2-glucose and α1,3-glucose residues from the oligosaccharide, respectively, generating monoglucosylated *N*-glycan Glc_1_Man_9_GlcNAc_2_ (stages 15–16, Figure 1). The monoglucosylated glycan is required for productive cycle interactions with the ER-resident chaperones calnexin (CALNEX) or/and calreticulin (CALRET) [11–13]. These interactions, associated with the CALNEX/CALRET cycle, facilitate folding of newly-formed glycoproteins in the ER (stages 17–20, Figure 1) [14,15]. If GANAB trims the last glucose residue it prevents further association with CALNEX/CALRET, allowing correctly folded proteins to proceed to the secretory pathway. In contrast, incorrectly folded glycoproteins can be reglucosylated by UDP-glucose:glycoprotein glucosyltransferase (UGGT) (stage 19, Figure 1) to ensure its interaction with the ER-resident chaperones (CALNEX/CALRET), allowing another cycle of CALNEX/CARLET interaction. This enables unfolded substrates to go through multiple rounds of interaction with the chaperons of the cycle until the native conformation is reached, when recognition by GANAB (but no longer by UGGT) allows exit from the cycle and the ER (stage 20, Figure 1).

Following the trimming of all three glucose from the Glc_3_Man_9_GlcNAc_2_ core oligosaccharide attached to the polypeptide, ER, and Golgi α1,2 mannosidases (ManI) collectively cleave the α1,2-linked mannose residues from the oligosaccharide precursor and, thus, provide the substrates required for the formation of hybrid and complex glycans in the Golgi of eukaryotes cells. The ER members of α1,2 mannosidase in various organisms play an important role in targeting misfolded glycoproteins for degradation by proteasomes [16]. ER *N*-glycosylation events are crucial for the proper folding of the secreted proteins and are highly conserved in the eukaryotes investigated thus far [17].

To date, *N*-glycosylation patterns and *N*-glycan structures have been studied mainly in mammals, insects, yeasts, and plants [18], with seaweeds and microalgae receiving very little attention. Among the scant research conducted on glycosylation in microalgae, most studies on *N*-glycan structures were performed on green microalgae (Chlorophyta) [18–25]. The studies generally revealed the presence of glycans similar to those found in other more heavily researched species, mainly oligomannosides or mature *N*-glycans having a xylose core residue. Whereas, two reports concerning the investigation of *N*-glycosylation of the green microalga *Chlamydomonas reinhardtii* describe two different findings [24,25]; Mathieu-Rivet *et al*. 2013, revealed that the predominant *N*-glycans attached to *Chlamydomonas reinhardtii* endogenous soluble and membrane proteins, are of oligomannose type [25]. In addition, minor *N*-linked glycans were identified as being composed of mannose, methylated mannose and xylose residues [25]. However, Mamedov and Yusibov, 2011 [24], reported that the *N*-linked oligosaccharides released from total extracts of *Chlamydomonas reinhardtii* carried mammalian-like sialylated *N*-linked oligosaccharides [24]. It is also noteworthy that the *N*-glycosylation pathway of the diatom *Phaeodactylum tricornutum* photosynthetic microalga was investigated, demonstrating that *Phaeodactylum tricornutum* proteins carry mainly high mannose type *N*-glycans (Man-5 to Man-9) and a minor glycan population carrying paucimannose type [26]. It was also suggested the *Phaeodactylum tricornutum* possesses the ER machinery required for glycoprotein quality control that is normally found in other eukaryotes [26].

Red microalgae seem to have glycosylation pathways that are different from those of other known organisms, as was been concluded in a recent study by Levy-Ontman *et al*. 2011 [27]. This study described, for the first time, the structural determination of the *N*-linked glycans in a 66-kDa glycoprotein, which is a part of the unique sulfated complex cell wall of polysaccharide from the red microalga *Porphyridium* sp. *N*-glycans were found to be of the high-mannose type (8–9 residues), with unique modifications that included two non-characteristic xylose residues (one attached to the core and the other to the non-reducing end) and an additional methylation modification on the sixth carbon of three mannose residues attached to the chitobiose core.

The work presented herein is focused on the red microalga *Porphyridium* sp. This organism is a photosynthetic unicell found in marine environments. One of the characteristics of red microalgae is their cell-wall that is composed of sulfated polysaccharide capsules. During growth, the external parts of the polysaccharides are released to the surrounding aqueous medium where they accumulate, increasing the medium’s viscosity [28–30]. These polysaccharides have been shown to possess a variety of bioactivities, with potential applications in different industries, e.g., cosmetics, pharmaceuticals, and nutrition [31,32]. Our group has undertaken the challenge of exploiting the potential of red microalgae sulfated polysaccharides for biotechnological applications and the development of large-scale production technologies [31–36].

In recent years, a great deal of scientific work is being directed at creating a novel assortment of pharmaceutical products using algae as cell factories [37–40]. However, although they are well suited for the large-scale production of recombinant proteins, the full potential of algae as protein-producing cell factories is far from being fulfilled [40–45]. Large-scale cultivation of algae for the production of therapeutic proteins has several advantages. Algae are simple to grow, and have relatively fast growth rate. In addition, algae are able to use sunlight as an energy source, hence they are energy efficient, have a minimal negative impact on the environment, and are easy to collect and purify. To date, the use of red microalgae as cell factories for therapeutic proteins has been limited by the lack of molecular genetics tools. A stable chloroplast transformation system [46] and a nuclear transformation system have been developed for *Porphyridium* sp. [47], the latter of which has paved the way for the expression of foreign genes in red algae, which has far-reaching biotechnological implications. However, the application of this platform cannot reach its full potential without the study of glycosylation. The differences in glycosylation patterns between different organisms may have influence on the activity of the recombinant protein or may influence its immunogenicity. It is therefore most important to evaluate the glycans attached to any recombinant protein expressed in any specific system.

There is very limited knowledge about red algal genomes; the sequencing of genomes of the unicellular red microalgae extremophiles, Cyanidiophyceae *Cyanidioschyzon merolae* and *Galdieria sulphuraria* have been completed [48,49]. In addition, only recently, the nuclear genome sequence of *Porhyridium purpureum* (referred to as *Porphyridium cruentum*) has been completed [50] and is the first genome sequence from a mesophilic, unicellular red alga that has been reported thus far. An analysis of the *Porhyridium purpureum* genome suggests that ancestral lineages of red algae acted as mediators of horizontal gene transfer between prokaryotes and photosynthetic eukaryotes, thereby significantly enriching genomes across the tree of photosynthetic life [50]. Moreover, based on the genome database it was suggested that red algae mediate cyanobacterial gene transfer into chromalveolates [51]. In addition, our group have made significant progress in the field of red microalgal genomics by the establishment of EST databases of two species of red microalgae, *Porphyridium* sp. (sea water) and *Dixoniella grisea* (brakish water) [32,52]. Non-normalized unidirectional cDNA libraries constructed from *Porphyridium* sp. grown under various physiological conditions generated 7210 expressed sequence tags (ESTs), which gave 2062 non-redundant sequences, containing 635 contigs and 1427 singlets [32]. Some genes derived from the EST database were analyzed and compared to other ortholog genes that exist in other organisms [32,52,53].

In this paper we describe our attempt to better understand the *N*-glycosylation mechanism that takes place in the ER within the red microalga *Porphyridium* sp. Our DNA scaffold (SCF) database of *Porphyridium* sp. was used to search for sequence similarity to algae gene products potentially involved in *N*-glycosylation pathways. Such a study can provide a basis for understanding *N*-glycosyation pathways in red microalgae, and lay the foundations for future gene cloning and characterization.

## 2. Results and Discussion

### 2.1. DNA Sequencing of *Porphyridium* sp

DNA was divided into sections of 330 bases (on average) and 38 bases were sequenced from each end of each section (Pair-end). The total reads identified were 38,537,782 sections, constituted of 1,464,435,716 bases.

Assembly of all reads was completed using VELVET; the best assembly results of the reads was obtained with a hash (or k-mer) of 23. Longer k-mers bestow more specificity (*i.e.*, less spurious overlaps), but lower coverage. A k-mer of 23 indicates more specificity on account of coverage. Nevertheless, we were able to obtain an impressive length of contigs, with N50 of 41,031 bp for the SCF (Table 1). The assembly results were also validated (Section 3.2.3). There were two types of assemblies (Table 1): (1) contigs containing sequences of the DNA reads only; (2) Scaffold (SCF), which consists of close contigs that are adjacent to each other using a number of unknown bases (Ns). Some contigs were joined by stretches of “N” when VELVET, through the paired end information, identifies a link between contigs but cannot determine the sequence. The length of the “N” was calculated from the average insert length. The quality of the sequencing results was high: 96.1% of all sections were mapped to contigs, while 83.7% of them were used for the contigs database and 89.1% were used to form the SCF database. The adjacent contigs were successfully attached to each other. Each base was sequenced, on average, approximately 70 times. The sequencing results indicated that the genome size of the algae (including its chloroplast and mitochondria) is approximately 20 MB. The genome size is in accordance with former results [50] and again demonstrates that red algal genomes are reduced in comparison to mammalian genomes [48–50,54]. Comparison of the genome size of *Porphyridium* sp. found in this study to that of some other previously reported microalgal genomes was found to be similar; e.g., the diatom *Thalassiosira pseudonana* (genome size 32.4 MB), *Phaeodatylum tricornutum* (genome size 27.4 MB), the green algae *Ostrecoccus tauri* (genome size 12.6 MB), *Ostrecoccus lucimarinus* (genome size 13.2 MB), and *Micromonas pussila* (genome size 21 MB) [55].

### 2.2. Identifying *N*-Glycosylation Protein-Encoding Genes in *Porphyridium* sp

Homology searches based on sequence similarities with genes encoding proteins involved in ER *N*-glycosylation pathway were conducted by using the TBLASTN function on the algae DNA SCF contigs engine database (Section 3.3.3). TBLASTN was selected because there is very little evidence for introns in *Porphyridium* sp. (based on our in house DNA sequence, unpublished results). In order to identify *Porphyridium* sp. gene products involved in the ER *N*-glycosylation pathway, homology-based searches of *Saccharomyces cerevisiae* (*S. cerevisiae*) *N*-glycosylation genes against the *Porphyridium* sp. DNA SCF contigs engine were conducted. The identification of the calreticulin encoding-gene in *Porphyridium* sp. was based on homology-based searches against the *Chlamydomonas reinhardtii* ortholog gene, and that of UGGT encoding-gene was based against *Galdieria sulphuraria* ortholog gene. Searches for encoding-genes of OST 3/6/5 and SWP1 were based on homology-based searches against the ortholog genes in *S. cerevisiae*, *Chlamydomonas reinhardtii*, *Galdieria sulphuraria*, *Cyanidioschyzon merolae* and *Arabidopsis thaliana*. Homologs were found for almost all algal *N*-glycosylation protein sequences in the ER pathway because all of them exhibit similarity values of above 43% under good sequence coverage calculated as compared to the entire gene sequences (above 60%), with one exception (sequence coverage of UGGT was only 26%) (Table 2). In addition the conserved domains that are essential for enzymatic activity were identified in all our ER *N*-glycosylation pathway homologues (Table 3). The homology was also verified by the GO values that were obtained by Blast2go program (data not shown). The predicted amino acid sequence for each gene was identified (Table S2).

All the genes encoding proteins involved in the biosynthesis of dolichol pyrophosphate-linked oligosaccharide on the cytosolic side of the ER were identified in the genome of *Porphyridium* sp. (Table 2). The sequences of these predicted proteins (Table S2) are highly similar to the corresponding asparagine-linked glycosylation (ALG) orthologs of *S. cerevisiae* (above 47% similarity, Table 2). Putative transferases, which are able to catalyze the formation of dolichol-activated mannose and glucose required for the biosynthetic steps arising in the ER lumen, were also found (dolichol-phosphate mannosyltransferase (DPM1), dolichyl-phosphate beta-glucosyltransferase (ALG5), Table 2). Almost all the genes involving the ER lumen biosynthesis exhibited above 43% similarity to ortholog genes. However, the subunits OST3/6/5 and SWP1 of the OST, did not exhibit homology to the related *S. cerevisiae*, *Chlamydomonas reinhardtii*, *Galdieria sulphuraria*, *Cyanidioschyzon merolae*, and *Arabidopsis thaliana* subunits. The STT3 protein (that was also found in the *Porphyridium* sp. genome), accounts alone for OST activity in some organisms [56]. In complex organisms the OST works as a multi-protein complex, built as an extension of the STT3 core [57]. Each subunit in this complex has its role in the fine-tube glycosylation process. For example, OST1 acts as a chaperon to promote glycosylation [58–60]; OST3/6 exhibits oxidoreductase activity and binds to specific proteins [61]; and, OST4 was found to be involved in OST3/6 attachment to the OST complex [62]. However, in some organisms, it is known that some of the subunits are not crucial to the OST enzyme function [56,57]. For example, the OST of some protists is composed only from WBP1, STT3, OST2, OST1 [56], or only from STT3 homologs [63,64], bacterial and archeal OSTs are composed only from STT3 homologs [65–68]. Indeed, based on these reports it is possible that the OST enzyme of *Porphyridium* sp. functions without OST5, OST3/6, and SWP1 subunits. It is also important to note that we identified two STT3 copies in the *Porphyridium* sp. genome (Table 2). These multi-spanned sequences have similarity of 66% and 67% respectively with the *S. Cervisea* STT3 subunit (Table 2). It is known that some eukaryotes, bacteria and archea extend their glycosylation ability by the duplication of the STT3 gene and diversification of STT3 specificity [56].

Genes encoding for proteins involved in the quality control of proteins in the ER were also found in the *Porphyridium* sp. genome (Tables 2 and 3). Indeed, Glucosidase I, as well as the subunits of α and β of glucosidase II, were identified (Tables 2 and 3). A putative UGGT and the two chaperons: calnexin and calreticulin, three molecules ensuring the quality control of the glycoproteins in the ER, also exhibit high similarity to ortholog genes (above 48% similarity, Table 2). In addition, three homologs for ManI enzyme were found, all belonging to glycosylhydrolase family 47. The three homologs of ManI were analyzed with InterProScan. The results of this analysis clearly suggest that ManIa (Table S2) is an ER enzyme while ManIb and ManIc are Golgi enzymes, harboring signal peptides at their *N* termini targeting them to the Golgi (Tables 2 and 3). This ManIa gene, that is known to be conserved throughout eukaryotic evolution [69–71], probably plays an important role in targeting misfolded glycoproteins for degradation by proteasomes in *Porphyridium* sp.

Taken together, these results suggest that the ER *N*-glycosylation pathway is conserved in *Porphyridium* sp., as in other organisms (animals, plants, yeasts, *etc*.).

### 2.3. Bioinformatic Comparative Study of *Porphyridium* sp. Protein Sequences Involved in *N*-Glycosylation, with Ortholog Sequences of Various Organisms

**Figure 2.**
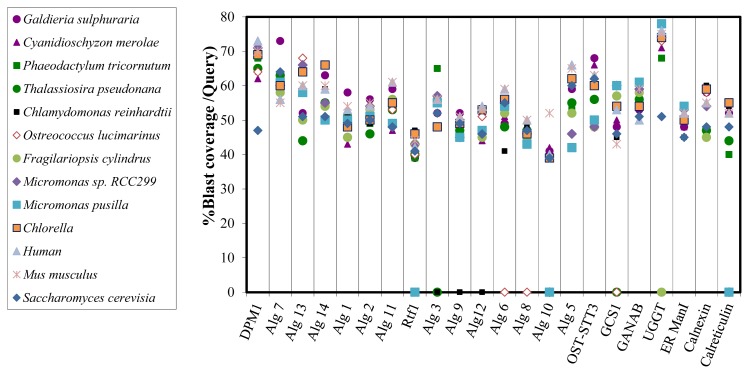
Similarity coverage (% positives blast coverage/query) of protein sequences involved in *N*-glycosylation from various organisms to the sequences of the ortholog protein from the alga *Porphyridium* sp. The results were obtained by BLAST 2 seq. Red—Red algae, Purple—diatoms, Green—Green algae, Blue—mammals, Black—yeast.

TBLASTN, a bioinformatics-based similarity tool, was used to compare between protein sequences involving the ER *N*-glycosylation pathway of *Porphyridium* sp. and ortholog protein sequences of other organisms. The various organisms tested included red microalgae (*Galdieria sulphuraria*, *Cyanidischyzon merolae*), green microalgae (*Chlamydononas reinhardtii*, *Osterococuus lucimarinus*, *Micromonas* sp. RCC229, *Micromonas pusilla*), diatoms (*Phaeodactylum tricornutum*, *Fragilariopsis cylindru*, *Thalassiosira pseudonana*), mammals (*Human*, *Mus musculu*s), and the yeast *S. cerevisia*e. It appears that most of the genes involved in the ER *N*-Glycosylation pathway in *Porphyridium* sp. also exist in other red algae, green algae, diatoms, yeasts, and mammals (Table 4). Most of *Porphyridium* sp. protein sequences that were studied, presented more than 40% similarity to ortholog sequences of various organisms (Figure 2). It is noteworthy that the similarity between *Porphyridium* sp. *N*-glycosylation protein sequences and ortholog sequences in other red algae is not significantly higher than the similarity found with other organisms. This can be explained by the theory that the red microalga *Porphyridium* sp. is an ancient organism that conserved its *N*-glycosylation genes. In a previous report, general EST-derived protein sequences of *Porphyridium* sp. were compared to ESTs of other organisms and the best homology was found to be the red microalgae *Cyanidischyzon merolae*, followed by the green plant *Arabidopsis thaliana* [32]. Looking at *N*-glycosylation genes; some genes that were found in *Porphyridium* sp., were missing in other green and red algae and in diatoms (Table 4). The genomes of several species of green algae, and all the diatoms, lack two enzyme sequences, α-1,2 glucosyltransferase ALG10 and GCS1. It is most likely that the genes are indeed missing from the genomes of these organisms—since their genomes are well understood. Further strengthening for this notion comes from the fact that these genes encode for enzymes which essentially act together—they are responsible for the addition and the removal, respectively, of the third glucose residue found in the ER *N*-glycans: ALG10 is responsible for the addition of the Glc α(1–2) to the *N*-glycan and GCS1 is responsible for trimming the Glc α(1–2) residue after the *N*-glycan is transferred to the nascent protein. This assumption was also verified by comparing between the similarity of the *Porphyridium* sp. STT3 sequence to ortholog genes of organisms that did or did not contain the ALG10 and GCS1 genes. Since the subunit STT3 is accepted as a substrate of the ALG10 enzyme, paucity in glucose residues on the substrate can change the connection to STT3. Indeed, the similarity between *Porphyridium* sp. STTs gene to organisms that were found to have ALG10 and GCS1 was higher compared to organisms that lacked the ALG10 and GCS1, indicated by changes in STT3 subunit affinity.

Based on the strong similarity of *Porphyridium* sp. encoded-genes to ortholog genes of complex eukaryotes, and the resemblance of *Porpyridium* sp. OST complex to that found in lower organisms (including prokaryotes) it appears that the red alga retained genes from both partners, thus, bringing together mutual elements in the red alga genome. Indeed, the *N*-glycan structures of the cell-wall glycoprotein within the *Porpyridium* sp. polysaccharide were also found to be composed of prokaryote to multicellular organism elements [27].

**Figure 3.**
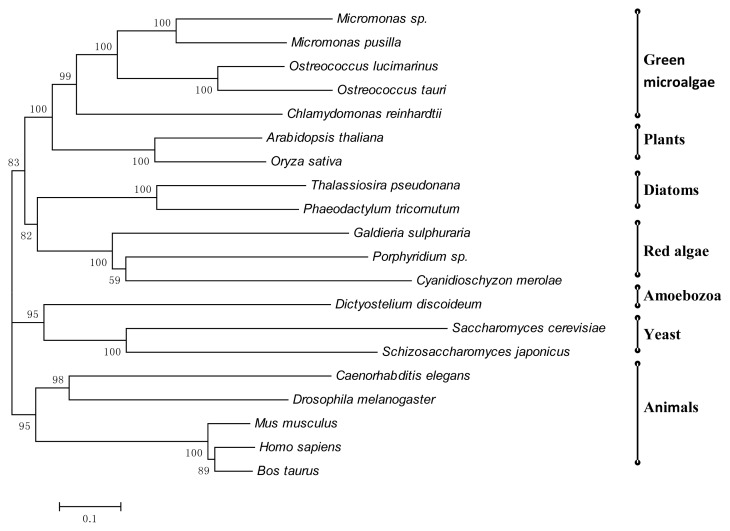
Phylogenetic tree of *N*-glycosylation enzymes (ALG1, ALG7, GANAB) of *Porphyridium* sp. The tree was built by the Neighbor-Joining method with 500 repeated bootstrap tests, using MEGA software, version 4.0. The conserved domain of the related enzyme sequences were tracked by Interproscan (1369 sites).

The sequence of the conserved part of three *N*-glycosylation enzymes of *Porphyridium* sp. (a result of sequence integration of ALG1, ALG7, and GANAB) was compared to ortholog parts of other organisms—other algae, plant and non-photosynthesic organisms—and a phylogenetic analysis was performed (Figure 3). As expected, it seems that *Porphyridium* sp. *N*-glycosylation enzymes have the same ancestral origin as other red microalgae as was previously presented [32]. In addition, it seems that the red alga has the same ancestral origin as the diatom group. This finding is not surprising; indeed the diatoms, are believed to be derived from a secondary endosymbiotic process that took place around one billion years ago between a red alga and a heterotrophic eukaryote [72]. Green algae and higher plants also show a common ancestral origin (Figure 3). These findings are in line with the idea that the red algae is a sister group to green plants [73,74]. Moreover, according to phylogenetic analysis (Figure 3) all photosynthetic organisms have the same ancestral origin, supporting the theory that endosymbiosis took place between eukaryotes and a cyanobacterium, giving rise to all photosynthetic organisms [75–78].

## 3. Experimental Section

### 3.1. *Porphyridium* sp. and Growth Conditions

The microalga *Porphyridium* sp. (UTEX 637), obtained from University of Texas Culture Collection, was grown in 250 mL Erlenmeyer flasks, each containing 100 mL of artificial seawater (ASW) [79]. The algae cultures were grown at a shaking speed of 100 rpm and aerated with sterile air containing 2%–3% CO_2_. The growth temperature was maintained at 25 ± 3 ºC, and illumination was supplied continuously from above with white fluorescent lamps at a photon flux density of 90 μmol photons m^−2^s^−1^. Cells were counted with a haemocytometer using a Zeiss light microscope (Carl Zeiss, Oberkochen, Germany).

### 3.2. Sequencing the DNA of *Porphyridium* sp.—The Solexa Technology

#### 3.2.1. Producing Genomic DNA from the Cells

A 48-h logarithmic-scale cell culture (12−15 × 10^6^ cells/mL) was centrifuged (3000× *g*, 5 min, 4 ºC) and the pellet was washed with acidified water (pH 4) to remove cell-bound polysaccharides and then centrifuged (3000× *g*, 5 min, 4 ºC). The remaining pellet (~1 g) was frozen immediately in liquid-nitrogen and minced into powder with a mortar and pestle. The pellet was incubated with gentle rocking for 2 h at 65 ºC. Ten milliliters of CTAB buffer (CTAB—3% *w*/*v*), NaCl—1.4 M, EDTA (pH = 8)—20 mM, Tris-HCl (pH = 8)—10 mM, 0.1% β-Mercaptoehanol) were then added to the incubated pellet. The mixture was extracted by mixing it with a 10 mL solution of chloroform: isoamyl alcohol (24: 1 *v*/*v*) and then incubating the mixture for 5 min at 25 ºC. Later, the extraction mixture was centrifuged (8000× *g*, 10 min, 4 ºC) and the supernatant was collected to a clean vial. The extraction procedure was repeated 3 times. DNA precipitation was obtained by adding isopropyl alcohol (0.75 of the total volume) to the collected supernatant, mixing gently and incubating at −20 ºC for 20 min, until a precipitate was formed. The DNA precipitate was collected by centrifuging (15 min, 8000× *g*, 4 ºC), washing in 70% ethanol, drying by vacuum centrifuge and resuspending in 300 μL DDW. To remove residual RNA, the sample was incubated with RNaseA (final concentration 150 μg/mL), for 1.5 h at 37 ºC. Afterwards the sample was precipitated again by adding 3 M sodium acetate pH 5.2 (1/10 of the total volume) and cold absolute ethanol (2/3 of the total volume). The sample was centrifuged (10,000× *g*, 10 min, 4 ºC), and the resulting preciptate was washed in 70% ethanol, dried by vacuum centrifuge, and resuspended in 300 μL DDW. DNA concentration was determined by nanodrop and gel electrophoresis.

#### 3.2.2. DNA Sequencing

DNA extracted from *Porphyridium* sp. (that was produced from algae cells) was sequenced at Fasteris, Switzerland by the high-throughput sequencing method of Solexa technology with Illumina’s Genome Analyzer EAS210R version-GA IIx (San Diego, CA, USA) using the Pair-end method.

#### 3.2.3. Contigs Assembly

The assembly of the reads to contigs was done using VELVET Version 0.7.54 suitable for *de novo* assembly [80]. Validation of the results was carried out by MAQ-Mapping and Assembly with Qualities software, version 0.7.1 [81].

In order to receive two database sources, two types of assemblies were made: (1) contigs database source containing DNA sequences based solely on reads, and (2) SCF database source, made by close contigs that were adjacent to each other using a number of unknown bases (N’s).

### 3.3. Bioinformatic Tools for Analyzing DNA Sequences

Based on the fact that most algal genes have no introns, the partitioning of the sequences to ORFs was programmed with Glimmer v 3.02 [82], in the contigs database.

#### 3.3.1. Blast2go

Analysis and annotation of the genes and proteins were done by blast2go [83,84].

#### 3.3.2. Translating DNA Sequences into Protein Sequences

The DNA sequences were translated into protein sequences by a program available on the ExPASy (Expert Protein analysis system) [85].

#### 3.3.3. Identification of Sequences by Similarity Comparison Using the Scaffold (SCF) Database

Based on the *Porphyridium* sp. SCF sequences database (Section 3.2.3), a search engine based on homological sequences according to similarity, was created by BLAST using Blast Command Line Application program [86], which is a part of NCBI C++ Toolkit.

### 3.4. Existence of Gene Sequences Involved in the *N*-Glycosylation Pathway

*S. cerevisiae/Chlamydomonas reinhardtii*/*Galdieria sulphuraria/Cyanidioschyzon merolae/ Arabidopsis thaliana* were used as a reference species for sequence-based comparative analysis. Sequences belonging to the *N*-glycosylation pathway of these reference species were taken from Gene bank (NCBI) and used for searching for homological sequences according to similarity in the sequences of the algae’s DNA using the SCF search engine (Section 3.3.3). The sequences identified in this study were translated into protein sequences (Section 3.3.2) in order to set the start and end of each protein sequence (the first methionine to the end codon, respectively). Each sequence was analyzed by BlastP to ensure the appropriate annotation to the related *N*-glycosylation proteins. In addition, multiple alignments were performed using the ClustalW algorithm [87].

### 3.5. Locating the Conserved Domain in the Algal Protein Sequences Involved in the *N*-Glycosylation Pathway

A homology study of conserved domains in various related *N*-glycosylation proteins based on the algae genome was conducted by blast2go. Separately, sequence detection of conserved domains was performed directly by Interpro and the added GO’s (Gene ontology) [88–91], which was weighted to the sequence annotation.

### 3.6. Bioinformatic Comparison of Predicted Proteins Involved in the *N*-Glycan Biosynthesis with Ortholog Proteins of Various Organisms

Sequences belonging to encoded-genes involved in the *N*-glycosylation pathway of various organisms were taken from Gene bank (NCBI) and used for comparative similarity with the predicted ortholog sequences of the red microalga *Porphyridium* sp. The results were achieved using BLAST 2 seq [83,84].

### 3.7. Phylogenetic Tree of *Porphyridium* sp. Enzymes Involved in *N*-Glycosylation

A Phylogenetic tree showing evolutionary relationships of enzymes involved in *N*-glycosylation, was built based on integration of three major enzyme sequences involving *Porphyridium* sp. *N*-glycosylation pathway: ALG1, ALG7, and GANAB, and closely related enzymes from different organisms based on amino acid sequences obtained from NCBI protein database. The conserved domains of *Porphridium* sp. sequences were detected by Interposcan. The tree was built using the Neighbor-Joining method with 500 repeated bootstrap tests, using MEGA software, version 4.0 [92].

## 4. Summary and Conclusions

The *N*-glycosylation pathway that occurs in the ER involves a large number of proteins, the majority of which are catalytic enzymes that are relatively well conserved in different organisms. We have identified genes encoding 24 products implicated in the ER *N*-glycan biosynthesis in the red microalga *Porphyridium* sp. A bio-informatic analysis of the *Porphyridium* sp. genome revealed the presence of a complete set of sequences potentially encoding for the following proteins: those involved in the synthesis of the lipid-linked Glc_3_Man_9_GlcNAc_2_-PP-dolichol *N*-glycan; some subunits of the OST complex; ER glucosidases and chaperones required for protein quality control; and finally, the ER α-mannosidase I involved in the trimming of the *N*-glycan precursor into Man-5 *N*-glycan. As all the encoded genes involved in eukaryote *N*-glycosylation (before the *N*-glycan is transferred to the protein) were found in the *Porphyridium* sp. genome, it can be assumed that the final structure of the *N*-glycan in the ER before it is transferred to the protein, is the usual precursor Glc_3_Man_9_GlcNAc_2_ as known in other organisms.

A study of gene conservation in the ER *N*-glycosylation pathway in *Porphyridium* sp. was also undertaken. The similarity of protein sequences to ortholog sequences from red and green algae, diatoms, mammals, and yeasts was studied. The similarity was found to be relatively high (above 40%), indicating the degree of conservation of the *N*-glycosylation pathway, and its importance in eukaryotes in general, and photosynthetic organisms in particular. This study showed that the genomes of several species of green algae and all the diatoms lack two enzyme sequences, ALG10 and GCS1. Furthermore, it was found that the similarity of the STT3 gene of these organisms (several species of green algae and all the diatoms) in relation to *Porphyridium* sp. ortholog gene was smaller in comparison to the other organisms that were tested. As the ALG10 product is a substrate for STT3, it is likely that these organisms (diatoms and several green algae species) do not express the STT3 products as active enzymes. These findings indicate a close evolutionary relation of red algae to complex eukaryotes. Conversely, the OST encoded-subunits that were missing (that normally exist in higher eukaryotes) and the existence of several copies of the STT3 gene in *Porphyridium* sp. indicate its relation to lower organisms such as diatoms. This finding supports the theory that endosymbiosis took place between a eukaryote and a cyanobacterium as a single event that gave rise to all photosynthetic organisms. In addition, the grouping of *Porphyridium* sp. sequences with those of other red algae confirms that the homologs found in this study are in fact orthologs of the *N*-glycosylation enzymes that are also found in eukaryotes.

In summary, we demonstrated that *Porphyridium* sp. contains the majority of encoded-genes responsible for the *N*-glycosylation pathway in the ER as in eukaryotes organisms. Studies to elucidate the exact mode of action of these encoded-gene products are currently under way.

## Supplementary Information



## Figures and Tables

**Table 1.** DNA sequencing results using high-throughput technology by Solexa, produced from the red microalga *Porphyridium* sp.

Assembly	CONTIG	SCF
Total length	18,613,981	18,925,597
Number of contigs	9,653	3,002
N50	4,218	41,031
Undetermined base	0	280,103
Average length of contigs	1,928	6,304
Maximum size	37,208	204,033
Reads mapped	37,023,682	37,034,742
% of all reads	96.1	96.1
Reads paired	30,970,611	32,980,567
% of all mapped	83.7	89.1

**Table 2.** Similarity of *Porphyridium* sp. proteins involved in ER *N*-glycosylation to ortholog proteins of *S. cerevisiae*/*Chlamydomonas reinhardtii*/*Galdieria sulphuraria*. The similarity analysis was performed by TBLASTN algorithm.

Abbreviation	Enzyme/Protein	Min. *E* Value	Mean Similarity Percentage	Coverage *
DK	Dolichol kinase	5.90E–20	55	62
ALG7	UDP-*N*-acetylglucosamine—dolichyl-phosphate *N*-acetylglucosaminephosphotransferase	7.09E–91	62	91
ALG13	UDP-GlcNAc:dolichyl-pyrophosphoryl-GlcNAc GlcNAc transferase	4.56E–32	61	93
ALG14	UDP-GlcNAc:dolichyl-pyrophosphoryl-GlcNAc GlcNAc transferase	1.54E–36	65	69
ALG1	Chitobiosyldiphosphodolichol beta-mannosyltransferase	3.43E–68	47	92
ALG2	Glycolipid 3-alpha-mannosyltransferase	1.24E–77	54	91
ALG11	GDP-mannose:glycolipid 1,2-alpha-d-mannosyltransferase	1.40E–95	63	83
ALG3	Dolichol phosphomannose-oligosaccharide-lipid mannosyltransferase	1.38E–90	64	87
ALG9	Dolichol phosphomannose-oligosaccharide-lipid mannosyltransferase	9.39E–102	55	87
ALG12	Alpha-1,6-mannosyltransferase	1.48E–73	57	86
ALG6	Alpha-1,3-glucosyltransferase	9.10E–80	60	70
ALG8	Alpha-1,3-glucosyltransferase	5.89E–68	51	83
ALG10	Alpha-1,2 glucosyltransferase	8.49E–28	43	93
ALG5	Dolichyl-phosphate beta-glucosyltransferase	2.15E–69	69	70
DPM1	Dolichol-phosphate mannosyltransferase	9.25E–80	71	99
RFT1	Flippase	4.69E–31	47	79
STT3		0	66	98
		0	67	99
OST1		6.00E–39	47	70
OST2		2.45E–27	72	80
OST3/6	OST-dolichyldiphosphoryloligosaccharide-protein	-	-	-
OST4		-	-	-
OST5		-	-	-
WBP1		1.45E–62	53	83
SWP1		-	-	-
GCS1	Mannosyl-oligosaccharide glucosidase I	7.55E–93	45	93
GANAB	Alpha 1,3-glucosidase II	0	62	71
GANABb	Alpha 1,3-glucosidase II,beta subunit	1.36E–33	43	98
UGGT	UDP-glucose:glycoprotein glucosyltransferase	1.16E–94	67	26
MAN1a	Mannosyl-oligosaccharide alpha-1,2-mannosidase	1.41E–82	55	72
MAN1b		5.83E–66	49	78
MAN1c		1.56E–62	49	82
CALNEX	Calnexin	3.46E–92	53	88
CALRET	Calreticulin	1.91E–58	48	94

* The coverage calculated as compared to the entire gene sequence.

**Table 3.** Existence of the conserved domain of *Porphyridium* sp. proteins involved in ER *N*-glycosylation. Separately, sequence detection of conserved domains was done directly (Blast2Go *versus* Interpro scan and then added GO’s (Gene ontology).

Abbreviation	Definition	Domain Identification	Name	*E* Value
DK	Dolichol kinase	PTHR13205:SF8	transmembrane protein 15	6.70E–29
ALG7	UDP-*N*-acetylglucosamine—dolichyl-phosphate *N*-acetylglucosaminephosphotransferase	PF00953	Glyco_transf_4	1.20E–49
ALG13	UDP-GlcNAc:dolichyl-pyrophosphoryl-GlcNAc GlcNAc transferase	PF04101	Glyco_transf_28_C	1.10E–26
ALG14	UDP-GlcNAc:dolichyl-pyrophosphoryl-GlcNAc GlcNAc transferase	PF08660	Alg14	2.00E–72
ALG1	chitobiosyldiphosphodolichol beta-mannosyltransferase	PF00534	Glyco_transf_1	9.80E–11
ALG2	glycolipid 3-alpha-mannosyltransferase	PF00534	Glyco_transf_1	5.40E–32
ALG11	GDP-mannose:glycolipid 1,2-alpha-d-mannosyltransferase	PF00534	Glyco_transf_1	8.30E–23
ALG3	Dolichol phosphomannose-oligosaccharide-lipid mannosyltransferase	PF05208	ALG3	4.10E–143
ALG9	Dolichol phosphomannose-oligosaccharide-lipid mannosyltransferase	PF03901	Glyco_transf_22	8.50E–97
ALG12	Alpha-1,6-mannosyltransferase	PF03901	Glyco_transf_22	1.30E–19
ALG6	Alpha-1,3-glucosyltransferase	PF03155	ALG6_ALG8	1.90E–107
ALG8	Alpha-1,3-glucosyltransferase	PF03155	ALG6_ALG8	3.80E–85
ALG10	Alpha-1,2 glucosyltransferase	PF04922	DIE2_ALG10	1.30E–15
ALG5	Dolichyl-phosphate beta-glucosyltransferase	PF00535	glyco_transf_2	7.50E–23
DPM1	Dolichol-phosphate mannosyltransferase	PF00535	Glyco_transf_2	8.30E–34
	Dol-P-Glc phosphodiesterase	-	-	-
STT3		PF02516	STT3	1.40E–142
		PF02517	STT3	3.30E–133
OST1		PF04597	Ribophorin I	2.50E–19
OST 2		PF02109	DAD	1.20E–41
OST 3/6	Dolichyldiphosphoryloligosaccharide-protein (OST)	-	-	-
OST 4		PF10215	Ost4	7.80E–06
OST 5		-	-	-
WBP1		PF03345	DDOST_48kD	1.20E–87
SWP1		-	-	-
RFT1	Flippase	PF04506	Rft-1	9.20E–11
GCS1	Mannosyl-oligosaccharide glucosidase I	PF03200	Glyco_Hydro 63	1.50E–87
GANAB	Alpha 1,3-glucosidase II	PF01055	Glyco_Hydro 31	5.90E–282
GANABb	Alpha 1,3-glucosidase II,beta subunit	PTHR12630:SF1	Glucosidase II b subunit	2.70E–28
MAN1a	Mannosyl-oligosaccharide alpha-1,2-mannosidase	PTHR11742	Mannosyl-oligosaccharide alfha-1,2-mannosidase related	3.80E–131
MAN1b				7.80E–95
MAN1c				1.40E–98
UGGT	UDP-glucose:glycoprotein glucosyltransferase	PTHR11226	UDP—glucose glycoprotein: glucosyltransferase	2.30E–168
CALNEX	Calnexin	PF00262	Calreticulin	1.00E–147
CALRET	Calreticulin	PF00262	Calreticulin	2.90E–63

**Table 4.** Existence of homological protein sequences involved in *N*-glycosylation of different organisms by searching for the similarity level to the sequences of ortholog proteins in *Porphyridium* sp.

*N*-glycosylation protein	Red Microalgae		Diatom			Green Microalgae					Mammals		Yeast

	*Galdieria sulphuraria*	*Cyanidischyzon merolae*	*Phaeodactylum tricornutum*	*Thalassiosira pseudonana*	*Fragilariopsis cylindrus*	*Chlamydomonas reinhardtii*	*Micromonas* sp. RCC229	*Micromonas pusilla*	*Osterococcus lucimarinus*	*Chlorella*	*Human*	*Mus musculus*	*Saccharomyces cerevisiae*
DPM1	•	•	•	•	•	•	•	•	•	•	•	•	•
ALG7	•	•	•	•	•	•	•	•	•	•	•	•	•
ALG13	•	•	•	•	•	•	•	•	•	•	•	•	•
ALG14	•	•	•	•	•	•	•	•	•	•	•	•	•
ALG1	•	•	•	•	•	•	•	•	•	•	•	•	•
ALG2	•	•	•	•	•	•	•	•	•	•	•	•	•
ALG11	•	•	•	•	•	•	•	•	•	•	•	•	•
RFT1	•	•	•	•	•	•	•	•	•	•	•	•	•
ALG3	•	•	•	•	•		•	•	•	•	•	•	•
ALG9	•	•	•	•	•		•	•	•	•	•	•	•
ALG12	•		•	•	•		•	•	•	•	•	•	•
ALG6	•	•	•	•	•	•	•	•		•	•	•	•
ALG8	•	•	•	•	•	•	•	•		•	•	•	•
ALG10	•	•								•	•	•	•
ALG5	•	•	•	•	•	•	•	•	•	•	•	•	•
OST-STT3	•	•	•	•	•	•	•	•	•	•	•	•	•
GCS1	•	•				•				•	•	•	•
GANAB	•	•	•	•	•	•	•	•	•	•	•	•	•
ER-ManI	•	•	•	•	•	•	•	•	•	•	•	•	•
UGGT	•	•	•				•	•	•	•	•	•	•
CALNEX	•	•	•	•	•	•	•	•	•	•	•	•	•
CALRET	•		•	•		•				•	•	•	•

• Homolog sequence exist. Gray squares: homolog was not found.

## Abbreviations

Annotation and abbreviations of proteins involved in *N*-glycosylation is detailed in Table S1.

ER: Endoplasmic reticulum
SCF: Scaffold
