# Supplementary material for: Genes Involved in the Endoplasmic Reticulum N-Glycosylation Pathway of the Red Microalga Porphyridium sp.: A Bioinformatic Study
Source: Int J Mol Sci. 2014 Feb 7;15(2):2305–26. doi: 10.3390/ijms15022305 (PMC3958852; doi:10.3390/ijms15022305)
Supplement: Supplementary file 1 [file ijms-15-02305-s001.pdf]

# Supplementary Information

**Table S1.** Annotation of proteins that are involved in ER *N*-glycosylation pathways.

| Abbreviation | Definition |
|---|---|
| DK | Dolichol kinase |
| ALG7 | UDP-*N*-acetylglucosamine--dolichyl-phosphate *N*-acetylglucosaminephosphotransferase |
| ALG13 | UDP-GlcNAc:dolichyl-pyrophosphoryl-GlcNAc GlcNAc transferase |
| ALG14 | UDP-GlcNAc:dolichyl-pyrophosphoryl-GlcNAc GlcNAc transferase |
| ALG1 | chitobiosyldiphosphodolichol beta-mannosyltransferase |
| ALG2 | glycolipid 3-alpha-mannosyltransferase |
| ALG11 | GDP-mannose:glycolipid 1,2-alpha-D-mannosyltransferase |
| ALG3 | dolichol phosphomannose-oligosaccharide-lipid mannosyltransferase |
| ALG9 | dolichol phosphomannose-oligosaccharide-lipid mannosyltransferase |
| ALG12 | Alpha-1,6-mannosyltransferase |
| ALG6 | Alpha-1,3-glucosyltransferase |
| ALG8 | Alpha-1,3-glucosyltransferase |
| ALG10 | Alpha-1,2 glucosyltransferase |
| ALG5 | Dolichyl-phosphate beta-glucosyltransferase |
| DPM1 | Dolichol-phosphate mannosyltransferase |
| STT3 | |
| OST1 | |
| OST2 | |
| OST3/6 | Dolichyldiphosphoryloligosaccharide-protein |
| OST4 | |
| OST5 | |
| WBP1 | |
| SWP1 | |
| RFT1 | Flippase |
| GCS1 | Mannosyl-oligosaccharide glucosidase I |
| GANAB | Alpha 1,3-glucosidase II |
| GANABb | Alpha 1,3-glucosidase II ,beta subunit |
| MAN1 | Mannosyl-oligosaccharide alpha-1,2-mannosidase |
| UGGT | UDP-glucose:glycoprotein glucosyltransferase |
| CALNEX | Calnexin |
| CALRET | Calreticulin |

**Table S2.** Predicted protein encoded-genes involved in the red microalga *Porphyridium* sp. ER *N*-glycosylation pathway.

| Enzyme | ***Porphyridium* sequence** |
|---|---|
| DK | GGAAGAAAVLFLLEVVRCASMPKVSPSLNVFMRALTDERDSGVLITTHMLLLVGCAGPLWVEDRISLHA<br>QSFASRSRALSGVIAVGVLDSLASFIGVHFGRTPWSAGTPKSVEGSSAGFVGAVACFYAIHCFTSSSPFAPI<br>PAPLSTRIPLMSSDIRSAAAVALASALAAVFEAHTTQIDNLVLPLYLHAALSLLL |
| ALG7 | MGAGWMYAGMLLVPAVGVLLRACIEAGDVYMVGVVARALLLAVAGMVLTARMVPAAQALMEKAQ<br>MFGYDINKKGTPQGAVKVPEALGIVPATIFIVILCLLHSHALRAAHAPAWVHSFLPLDAGRSSLLPHDDSN<br>LSSVVLYTSALASVTFMTLLGFADDVLDLRWRYKMVLPLFGCMPLLANYSGSTSIVIPVPLRSLLFDKTLL<br>DIGLLYFIYMALLSIFCTNAINIYAGINGLEAGQSFVIGCFILIHNAMQLNPHRHVEDLALLRSNNMFSVEI<br>MLPFVMVTLGLLRHNWFPSRVFVGDTFCYFAGMTLAMGAILGHYAEILLLFFIPQLINFVYSLPQLIGIVP<br>CPRHRLPKYNVETKKLEPIKSHLNLVNLTLLCTGPLTEKRLCEVLMLFQVACCSAGLAMRYLVIKYLL |
| ALG13 | MGASGERSVFVTVGSTEFDALIEAMTAPQMLRALRALGYTKLRVQYGRGKFVPRACGEDDGLFDEMVD<br>CFRFKPSLTHDMQRAGLIISHAGAGSIFEALRMGKNLIVVINDRLMDNHQAELAEEMQALGHSYAATCSN<br>LLDVIQSSVRSRCWRPFSVRSLGSPDTLRALLMVSHNVDKS |
| ALG14 | MSGQESDVGHVVSAALAAAVFLSVLTSLCLLASNNIRRRRKLVARTSSAEANMPPESLSLDPLPHACGNG<br>AGHKPTIMFVLGSGGHTAEMLHFVRDWFRCMPTFDENQRGIAYKFVFVVASTDNHSEQKVLELFKDAA<br>RGASHVEYEVAKIPRAREVRQSWVTSVFTTFIALLSSVIVYLKHRPDVLVCNGPGTCVPMCACALFGRAV<br>AVATTSVIYVESVARVRHLSLS<br>GKLLYWLVDRFIVQWPHLQQTHPLSEYHGRLT |
| ALG1 | MEWTITVFLWTCVALLCVHVAWGLDEIARLTWACLCWASRVGGPKIQEDLTRDVHVAVLVNGDIGQSP<br>RTLHHAEMLAKWSFRPSVSPSQFGAESDADLDEAGATVLSKGYYEGGAISQQGAVPRSSTRVTVFAYGD<br>PPKSLVQTEPGMPHPQFPTQGQTSPQVTFRKIHARTRVRRDHGSAVYFITSVLATVSRALDLIEALWTCRP<br>RLDALIMQNPPSIPSLLIALVF<br>ARLRHGALVVVDWHNFGYTILRTTRAPSLLLVLAEWYESTLAPYADAHLCVSTAMCEFLRDEWNVDAT<br>VYYDMPHCVFRPVSVEEEHDLFLRLEQEYSLPIATERGSYPVSCTLRTSCGERNQHTTRRSDRPFLCVSST<br>SWTPDEDFELLFDALVELDKSLLQMSYSNTRTGETQVSNLERSLVSPPQLLFVITGRGPLRAAFEARIARA<br>DLRCILVVCAWLSKQDYAHLLGSADVGVCLHSSSSGLDLPMKAVDMLGAGLPVIAVDYGCITELIVPGK<br>SGLLVRGADELASTILDLMDGFPKGKKRIGSEKGFGSLQVMREWIRAKYGSLDQRADSEWCRVVAPKLN<br>TLLDLREDLPDPPSKFSL |
| ALG2 | MVDEKEGRRVAIVHPDLGLGGAERLVVDAALGLQERGHKVVIYTAHRDVQRCFPEVRPRHLGGSVSCT<br>LVNTYVPRSVLGRFHALCTVIQCVILALYVCVNASRVDVAFCDIVSAPALIFRMFGIPCIFYCHFPDQMLA<br>DSIRVSHAKLAEVDSSFVAVIKRFGRLVYRAFVDRLERFAISRATCVAVNSQFTARMFSEVYPDLLRGHE<br>RKEPIVVYPAVNIRPSVGHDASLTSRLWATFAEEMFPIVKDPILLVSLNRFEKKKNVALAVRALHAIRSSD<br>SLVPNKQLRDSIVLLIAGGDDDRLEENKQTLHNVRQLISELRLEAFVGVELNFDESKRISILRAARALLYTP<br>SSEHFGIVPLEAMAAGVPVIAVDSGGPRETVQDQATGFLCPAEPAAFAQAAARLVVDQKLAEELGKRGR<br>ERAQSLFSVDALGTALEGLVHECCDAKLARTATKPSSRKKQS |
| ALG11 | MVLRYSYSSRVGQCRGRLDGTHEQECGDVFDVWVRPGGMEWTERVRAGVHVVQLGLFVLYVSVLLGF<br>GLLVLRAMRARQRRDREWLRRTRPSKDDASCAASGNAARDAKDAFVVSFLHPHALAGGGGERVLWLA<br>IKALRGRFPDATDLKIRVFTKGAPRKADVLEKVFVQFGIDIFTTQFELRELWSESLLDGARYPFCTLLLQFL<br>AGAIAGVECTLLHVATDLFIDTTGHAASLAVAKWLGGCHVATYVHYPTISTDMVHVVRSRTSQFNNSSR<br>IADSLFLSGAKLAYYRLFSHMYGLAGGCADVVMVNSSWTRSHISSLWSGSRRKISLVFPPIDTKPLLAFG<br>MDKRDPALIVSVAQFRPEKNHALQIEAFVKLLHKQSRDPRSDTLRPRLVMIGGCRNADDAARVELLLYEI<br>QNTYHLRVPDQVELLVNVTRDQLHAYLAKASAAIHTMKDEHFGISVVEFQAAGVIAVAHNSGGVALDII<br>QNGKYGFLAETADQFADCLSSALSLNSSSRLAMIQTARDAASRYSDEGFATEVLKALQRVLPVD |

**Table S2.** *Cont.*

| Enzyme | *Porphyridium* sequence |
| --- | --- |
| ALG3 | MARGTDGSLACDGLTRRQAMGVTAEHVGARIMCSKENERGRTQGGGFACASWTRMIGEVWLDRLDRYTKRRDGFMILSAFVLLFVAVSGSFVIVQVPYTEIDWVAYMQEVAGVLEGERDYIKLRGDTGPLVYPAGFVYIYSFLFYVTDAGVNVQLAQWIFLAVLLCTVAVVLGIYRVAITSQPGLMPPLVVMLLVASRRVMSLHVLRLFNDCVEALLAYASILLFAHNKWAFGCVLYSLAVSVKMNALLYAPALLMLLLQANGVARTIGYLSICAVVQIVLGLPFLISHPVQYLTKAFELSRVFEHRWSVNYAFLSIPTFTSKGLALVLLLGHLATLAWFGSREVWGRKVHPVRSTGRAAALDADYVLRVLFTCNLIGIAFARTLHYQFYAWYFHTLPYLLWRGRLPFALKLAVLLGIEMAYNIYPPRAWSSIVLHVCHAITIAALSATSSVSKSETNKHNH |
| ALG9 | MRRRRSGPGPRGGGSSSGRERETRDGVVDRKPGGTAGSQRRMRTALSAIFSRHRAVPGWAPNVVTAFLFLLFLRLGSAMVSGIEDCDETFNYWEPLHYLVFGYGFQTWEYSPQFALRSYVFLLPYSVVAKIGSMVSLGSKGPAEIKYNAFLAVRFAQAFACAAAETYLYDSVIWRFGKPAAXXLLLALLMAAPGLFRASVELLPSSFAMIGVCAATAAWLVGEFQLAVLGIAVAAVMGWPFAAVLGLPMSFHITYRKGILQFMQWMFSDGLVLAFTCFIVDTRFYGRFTLAPANLVIYNVLPAQGAGPTAFGVEDWKYYVFNMVLNLNVSALLLVLYPLLWIWDGLVADAWPTRQDALTRLIFLSPAFIWLFVMFNQPHKEERFLAPVYPLVALVSAVSLDDVLRIVFGLSRNLSESSRKYRVIVKNLFCLAVVCVAFALGASRMLAVIKGYSAPMKIYTHLSTLELQYGEGPRSKALPQDVYNICVGKEWYRFPSNFFLPSSQFRLRFIKSEFSGLLPKEFAESGRGTQRTPPGMNMYNEEDPAQYFNDTLACHYFVELDLEESVSGTTPTNPIPPEARAAIWEEDFLWSEKSRPFFRAFYVPGWEHQYWTLAKYRIYRNAHLLPFRRN |
| ALG12 | MRLYRALPFCWILAYVALAPCYQKVEESFNTQAVHDLLYHAWWRRDKIQQHFDHVAFPGIVPRTFIIPCVIAALAFPFRLLLGATGKRLVHLVARLVVGAASAWSLDLIAGALEASHGVVIARAFVVVSCAQFHALYYASRTLPNTFALILTNAALAMRIRRRFEASWILLAVVVALLRSEVVLLLICCLVVDLWPLKNHSLESLVRIALKLFSSALVTAFFSVAVDSYFWQRLSYPELEVFYFNAVLNKSSDWGVSPFHWYFTSALPRALAGAFPLAAFGLVQDPSCRRTCVPFVMFVALYSFLPHKELRFIFYAIPACNVCAATTVATVWTGRSSSKQSRLAWIMLLGILLVSAALLPLYASASYWNYPGGRALDGLIHGSAECEGNSTRGPLRVHIDAKSATTGINRFLERDDGKWLYSKEEDHSILTWVSDFELLVTERPTVDGFMIIHEEPAFRRLRFPKALSWAELSAVVAVEPAIFVQCNTALHATCENLQACFDVKTSGRASRTISGEAKEL |
| ALG6 | MEDETSARTNTEAELARAVTRVALDGSASAMPVGPRRGALAVITLAAVLWRSLVAMYPYSGEGLPPMYGDFEAQRHWMELTVNLPPRMWYVESELNDLKYWGLDYPPLSAYMARLFASFAPPESVALVTSRGFESEVFRAWMRNSVIVADLLVWFPAVALFVSTYXXRTAAERDETALFAVLVAMPCLVLIDHAHFQYNAVGLGLFVLSVALLLRDSLVPDALGCFAFCLALNFKQMNLYYALAVAFYLLGKASQRLRASGLAHAFTYLLILAGAVFVTFASVWWPWLGAWDDVRAVLLRVFPLHRGVYEDKVANAWCSLGLFYRPLRRVTPWACLCATLLASAPFCLSVLTKPGRRTFVLACAGVSLSFFLFSYQVHEKHVLLPLTAVTMLCSDAPWLSVWMNAVAMLSVFPLLDREGSSLAYVGSQLLNVCVHLFFFRGIDDSMPPSKSTSSSSSSSAAAAAGHRWRAVAVTCHIVACHAAYELVRRLPCLIKRWPDLPTYVVTVSSFLHLCAMYVVLLRLLPRLPHAQHRYSSSAGTAEYSITEPQPLKLG |
| ALG8 | MDGARELLPLAAREREEEYHESRTLVELAKGTTKPVYIMARVTLLDPTLASLLTLSVALRVLLLHARYASTDLNVHRDWMALTWNVHVRDWYTAEISQWTLDYPPLFAYLEYALAGVAHVLQVPGFELEDAGTQVNASATVFLRSTVLVLEILFLFSGMYVLVGSLYHDRGADAATAPSVTQSSAATMALSLGWLSPGILMVDYMHFQYNSIALGLLLWTCVLLGNERKRLALDVGVALFVTALNTKHTLLYVAPCIGAAVLGVSFNAGEDLKSAWSLLQRCLTLLRLGVVGTVCMLAIWSPFIYHGQIAHVLMRMFPFQRGLLHSYWAPNLWALYAGTDKLLAFVMGSEREKAWSTRGLVGVMHPFAVLPSVGPRACSALSLAAMIPAILLMLRLGNLRKSCFHGKTRLGIVLFSTAYCALCSFVFGYHVHEKAILLCVIPLAPLSTLAPEYMRIFRILACAGYYGLLPLLFTPAEQVPKCAFFVLHSVYLHRVSQKMQVNESAGEWLYMRGFVVLELYCQFVHTWWFGHDRMHFLPLMLVSMYAACGVLVAWAMGLGRLFALHCELMAQNGPKSAKSE |

**Table S2.** *Cont.*

| Enzyme | ***Porphyridium* sequence** |
|---|---|
| ALG10 | MPQPALVAAGAMLLVCLCLISALQPKPYMDELFHVAQTQAYCRALASSSPSNMLDAIKSTPYDPAISTPP<br>TPYLLVSVVVRYIVLAPFPRLVPVLCSVPALRIASACIAFCALLQVHAILKNVLIKTATRQELLHHYLRIGM<br>SQADWLSALALWLHPISSFFYMMFYTDNLAMLFLLLCMRQSHVFREPTLGKPVRVEYVAALMGVLASS<br>VRQSYMIWHALVVACSVVALAEEMHPKIGRKTLQVERMWAHRATITWILWPHVLAGLLYAGFVAFNG<br>GVAIGHREFHQPQPHWMMFWYFCAYRLIFPYPDACRAEDVTVAGFSRAYLFSALEGTNARNRARMVVL<br>ANLVLAGLVIVSIWLGTIVHPFVLADNRHYSFALFRRVLTPSARFLFLPTYMLGFWVLVADLGPFCIATLP<br>LLALGLVPVPLFEPRYFAPPFMISQFLTLLTQSRAARQKWYCGWFSSGIALSQIAAMLALFFFVPFSRPPDA<br>HLPQDASLGRWMP |
| ALG5 | MLIGWVAAGKVSRLRISRESVRTAQYLCWRRLRTGQIRATPCLQVRRRCRYLGTSADRRPKLLGQAEGE<br>GNMSSNIPMSEYTYIDPASMRPGRKEMFPSLFSSPAEVRLTVVVPAYNEEARLPAMMDEALAFLEKWGE<br>EDNSFTYEIIVANDGSRDKTALVALEYTKRFSAQKVRVLSLAQNAGKGAAVKKGIMAARGAVILFCDAD<br>GATRFADLSILYRQLELIARDQQADSLENAHACVIGSRYHLKSSAERSLVREFVSRVFNLYVQYVGGVRG<br>VRDTQCGFKLFTRRSAQLIFPCMHLDRWAFDVEALYIAQAHCVAISEVPVQWMEIPGSKLSVVKASLNM<br>ARDMALMRWNYLTGVWSAGVPDLAHVGTQSNKFP |
| DPM1 | DGLLVLSDTRMAGSDGPRGRDLYSVLLPTYNERENLPYIVWLLVRAFRSAGERCEILIVDDNSPDGTQEV<br>ARRLQRYYNRHDADSESDGLQDDVRIELLTRAGKLGLGSAYMHASKQARGNFVLILDADMSHHPKYIPR<br>MIATQRTADYDIVTGCRYVPPHLGGGVHGWDLRRKLVSRGANFLAQLLLRPGVRDLTGSFRLYKRSAFE<br>RIMQHMRSSGYVFQMEIIVRARRLNCSIAELPITFVDRLFGTSKLGSLEIVEYLQGLWMLLTS |
| RTF1 | MGHDETGEGQDAALGLQQGEHRHVAFVGAAMRGLSVIVLLQFVARVLSFLLKVVCARALGPARFAFGE<br>VKLQLLVALALLPAREGFRKVALRARSDAHAAMLSWTAAAASCLIAVLAWRLFERFGLSHDLDAPDRY<br>VHSLALMVAAWAAAIEGVAEPSVVACARYQLYTAQAVSKSAALIAASCVTVVGVYRLPEVYLVLASAF<br>GLLCYALLFLLFLFFAVWRHEQAQAATAPRFVFCSPFRTFSATPSGRDDGVIIVQQLYQALVRFALGDGE<br>NFVLLVTCSEQEQGAFKLASNIASLIARFLLEPLEELCFNVFSRLGNDLASFPQPTPGGSVTRVGSKTRDNS<br>SAFHTLETTLRVALTVVVLTTGMVACIGPSFATLFVHLMYGSTWAEHTRAPMLLSMYFSYVMVMSVNG<br>VVEALLNATATQKQQRSYAAFTTLVSVGYLAAAWVSSSHVLVGAAGLIMSNAVNMVLRIMFCARYAL<br>HFVHMPLGWLGVIFPRARSGAGLALCGALTFSARRWLLPAVDTAKSGVALLLSPAVCAHFLLGVCSTAG<br>GLGWIYTCERETISLGLGLYRGRGRGDETSKHL |
| STT3a | VDDGTYAFWNWFDASSWYPLGRIVGGTVYPGIMYTAALLHRAYRVIGIDLDIREVCVTLAPVFSGITALA<br>TYMLTQQTWNEAAGLLAAAFVGIVPGYIARSAAGSYDNEAVAITALILTFALFVKAVNTGSIAWAALAS<br>LSYLYMVSSWGGYIFVMNVIPIYVLTLLLMGRYTNRLYVSFCAFYVLGTLLSMQIRFVGFNAVQSSEHM<br>GALGVFGILNLYCCAMWIQSFSSPQTFRAVLRLLLMGALSLAGIAAVYGIYSGYIGPWTGRFYTLLDPTY<br>AKRKIPIIASVAEHQPTSWSAFFFENHFLVMLMPVGIYHVLRNPNDTNVLLVVYGVFSTYFTGVMNRLML<br>VYTPMCCVLAAIAISELLSVWMVPLKQKGVIPSVRSLFRSDVSSEEAASTSAAGSTSRKTAKRMSKRDAS<br>SSTAVQQHGTAPVTDQVEVSLGLILVVFGMGIAFVHHCVWSSSEMHSSPSVVLSYKVRSGDRVFIDDFRE<br>AYQWLNQNTASSTRVLSWWDYGYQLAGMSNVTTIVDNNTWNNTHIGTVGRCLNSDEVVAHRIARKLD<br>VDYVLVVFGGLIGYASDDLNKLIWPIRISGSVDPSVNERDYLTANGEYSIGDDASETLTNSLMFRLSYHRF<br>ADVVAPSVESPIRDQNRGTVSKKARDIRLHSFEEVFTTGHWLVRIYRVKPPHARGFPLLAPVAET |

**Table S2.** *Cont.*

| Enzyme | *Porphyridium* sequence |
|---|---|
| STT3b | MAREGLAIRQSEALVRLGTMALIYVMAFSARLFSVIRYESIIHEFDPWFNYRSTKVFVEDGMYAFWNWF<br>DHKSWYPLGRVVGGTVYPGIMFTAGFIYHALHALGFPEIHVREVCVLTAPIFSGLTAIAAYLLGTEAYSSG<br>AGLFAAVITSIVPGYMSRSTAGSFDNEGVAITALVFVFYGFMRAVRTGSILYSALSAIAYLYMVSTWGGYI<br>FVINIIPIYVMVMLVLGRFSNRLYVAYSTFYVLGTLLSMQIRFVGFGAIQSKEKLAALAVFGFLHLYVFGR<br>WLYSLMPRRKFWLLFSGTVAVLVGTVAIALSWAFRTNFFGPWEGRFYTILDPTYAQRFIPIIASVSEHQPT<br>AWASYFMDLHVLNFLFPVGLYYLMKGVTDTNLLLIVYAVFAAYFSGVMSRLMLVLAPASALMSGVALS<br>EMTNKAAASVFEMVKRAPHDGSSTSSTATLHPDGGTASSTDTAAQSGKRGAAKKAVATRKAASVQGSA<br>STASSSGKTGAAMSRPFKVTLEVSVALLLIASFVLFKYIQHCLYMANHYYSSPSVVIQLNDGSYWDDFRE<br>AYFWLSQNTDPDDTVLSWWDYGYQLSGMANRTTIVDNNTWNNSHIATVGRCLNSDEKKAHTIARKLD<br>VDYVLIIFGGLVGYSSDDINKFLWPIRISGSVDPSVKEEDYLTARGEYSMGEDASETLKNSLMYRLSYYRF<br>NEVRNHGNFAVDLVRRVQAPEHDITLRYFEEAFTSEHWLVRIYRVKQPDALGFT |
| OST1 | MWECISTFPARSVITNRRLLPFFCAFLISLRDLCFIGLFLARFGHFQKLDKMVLTMMVQCTVQNQFPDQSI<br>AAYRVAIHPSDAEALHFLQACEHTDCFTDADGQRRLLSKTVEEGRDHGAQLYSFALTEPLMPGEERTLII<br>KYGFGQALKPVPESNVQTSKQVLKFDVSSEFFSPYVTLQDALELKTASGWTIDAVRSDSNDVKKISTGIVS<br>RNVLAGVQPYTYTPVRVLVSGNSPLLKLDSFRKIFTVSHWGNVNVREEYDLRNFGTALRGQNSRVDYDR<br>GQHFNSVPKLRFRLPPDASNVYYRDWDGNVTSSTLHKPGVRTRIFDATLRFPLFGGWKNAFWISYDLPAS<br>SLLSQSVGESTRFQLVGIVAPTIDASSILIDDLVVAVSLPEGSHTHDAFVNGLDVETIDFGRNPATLAFKGR<br>PVLELHMGTVLTGLSVAPSVVVEYRFSPLSLLLGPFMIISFILLGFVAWILLGYMSDVLIITPADAVRMTHP<br>VYAKEKGQFAALCEGVMRVSAALHTLAAGISIPDQLQFFEEKSHALVLELGALRSAVKSLEADVASPVFF<br>THVSNLVDL<br>YNEWIPLKEQQVRREPMAGDRLRELDGCLELELADLKFALGSI |
| OST2 | MASSSVSSGSGSGSGPKSVWGSLKSGYEAGVPLYL<br>KVIDAYILAVFMTGIVQFAYCMVVGTFPFNAFLAGFISTVGTFVLTVSLRMQVNPQNLADPANSWQSLTL<br>GRVVADWLFANLVLHMTVLNFIG |
| OST4 | MITDEQLVSVATWGGYLVIGLVILYHFVVASASALPPSASASSSASTKKDL |
| WBP1 | MRLQNTWCGPMAGSFWYRLQPRGSTRWVLTMLGMIFMLLIVMGGAGVVAIGPDGRDRVVVIVPTLSD<br>MQEKYKTIQEHLVETGYAVTVKALDAPDTAEILLMQDGEYVFDTAVSLVPRAQNLGPGWSAGAVLDFV<br>DQGGSVFVAADYNYGAFTKQLAAGLGVQLDDKLNVVIDHGSFDAGLDQDGSHSFIKAGGVTKAKPIVN<br>AGSPSASSILFKGVGASLYTSNELVEPVLWGSPSAYCGRKFESATDIPLASGNEVVLGAVLQARNTGTGR<br>GAYIGGVAMLQDQVMQLAGVKHRDFYLDLLSWTCGERGVLKAENVRHWLANNGEQRGTYKVQDDIG<br>FALDVFEWAGALGHWIPTTPEDMQVEFTMLNPYIRARLVPLVSESSGESASMHANLTIPDVIGIYKFEIAY<br>VRTGYSHVALMENVNVRPFWHNEYERFIPQAYPYYASAFVMMGSLIVFTAVVLYGKPTVDAERQHKAK<br>DAR |

**Table S2.** *Cont.*

| Enzyme | ***Porphyridium* sequence** |
|---|---|
| GCS1 | MDTDTAVYTRSTAVCINASSRRASRHTASPTASLLTTYAIISRECSACSSCASGAPPRAPSASILLPLLFPFR<br>KLVDDQRSSRQALGRDVSTRLRRACRMGMARRACAVRVIALLAVLVGARSCVLADTPPNHRRWGLWR<br>PRLIAGVRSNVRDSAMFGIGWQGEGAVRSALRMCADDGSNGVQFGYVRHDGRAYAQQVIVDAQLQVK<br>MNMDWILVEHKTRDHLPAFAWVLRITGEHIETEQDSRSASGASYVSLFLTAASGADEDDIEGQEEEPHVL<br>SEIECTGHSSDVCIQGQSGRSVSALTPYKLMYKQPTYGTPATLSFAARPPKLAYPEHIVSTWDASISALLV<br>AKDASKHSKGPRRDLSQPVTVLGAWAREQQFASEQHVLRYMKDDGSGVRTLHADDPEAVDSCADART<br>CSVAVVQRVLERDFRVEIVFSEFAFDDELLVSLCGAALDERIERARRAFDDRFHALFRGIAQNYKAGSTET<br>RMATYALSNLLGGFGFFHGSSWVERENTEIATSTDAGIEPKNKLETREVAQVLGQDGKLAALTPQNLFTA<br>TPSRVVFPRGFLWDEGFHQLVVLQWDTQLALESLMSWLGVIRSSGWIPREQVLGFEARAAFPKHISHLMI<br>QNPSVANPPTMLMPWQVLARRCHNMHQGTKENSLDRVHPEQDATCSAATWEHVANALGLHLRWLDT<br>NQRTRDASAYQWKGRNERHRPQNGRNPFTWASGLDDYPRARVPSAQEKHLDLHTWMVWAHAAMVTI<br>TSLAGHSESSVDALKRRADELKSMMETQFGGGSDRHGLLFDLDRDGAQIEHVGYVSLFPLMLGVLPHDS<br>PRVGAALAAMQDPEQLWSVAGIRSLSKSDDYYLKGDQYWTGPVWIPINYLLLGALHNKYAAYPGPYRE<br>RARALYDDLRRTIVTNMARNFEQQSTLFENYNDRTGDGQKGRLFTGWSSLIVLIMAEEYEGLIV |
| GANAB | MRNAARGRALAARALLWVAGVLALAILAFNPLRECEGATWNKLKTCAQSGFCSRHRGLPPRPHNQVTY<br>AVRPESVHVGSSPNDDGAVSGLVSISTVDSGSEAESMSVDLAFQIRAYDNGVMRWTLDEQPGNGRFERY<br>RPTDGVLVDSLRAVRITEDTDLDRSSPLRLRVRCSACKAGLTDPPVLVIDYNPLRVTLESARGAPLVILNG<br>HELLRFERQDEDIIEPPESQQHEEAPTHQANSEHSPEVGGDHEGASHVADGTDHSDEYMAGYYDDVAGN<br>ENDFGLDAYTAPYEDYNHGLDDMHAVPYGEDAVPDDIALREFEAPVAPHGSETAHACRGCFQETFDGH<br>TDVKARGPESIGVDIEFPRASHVFGIPERTSSFALQDTKRDGLAQSGESLSDPYRMYNLDVFEYELNSPFG<br>LYGSVPLLTAVTDGGHWSGVFWLNPSETYVDVTGANGTASGGHNGSNTSITTHWFSESGVMDVFLLGG<br>GDMPCIVYNQYVSLTGPAAVPNTFALGFHQSRWKADFEADTRAVDRSFDTHDVPYDVLWLDIEHTDGK<br>RYFTWDLNKYPNPVQLQHDIDARGRKMVTIIDPHVKRDGNYALHRFAEENGLYVKEADGTTDYVGFCW<br>PGSSSYFDFVNPAVRAAWASRFSPEFYKELTPSLYTWVDMNEPSVFNGPEQTMPKGLKHFGGWEHRDV<br>HNLYGLFVQRATFEGLLQARNSTDRPFVLSRAFFAGSQRFGAVWTGDNAASWGHLQASIPMLLSLQISGI<br>VFSGADIGGFFGNPTRPLAVRWYQAAAFQPFFRAHKHIDADPREPWLLGNDNMQHIRKAISERYTFLPY<br>WYTLFAVASTVLDASDARAASKDGMHHPPMRPIWWHFPSERALLGGKEQEHSWMVGDALLVAPVLSE<br>NTEAHRVRLPGGGGGDVSKNAPKSANSNSGATASRWFDLYGDYAELSGGETHIYNEVSLDRMFVFQRG<br>GTIVPRKMRRRRSTVAMNLDPLTLVVALDSFETAHGTLYVDDGKSFAYEEGNFVVRSFEFSSNRLTARTV<br>AGSEEWLDDQRTERSRILCEKILVLGLAVEPSTILAETIHRNADGSYVPKTVELERDINFNFYAQSRKLVV<br>RRLPFRAYSGDWTLHLM |
| GANABb | MARELLGLRSACHDSRVLLAAAKDMRALQLSLSAVVAVVLAAHALSRVHGDPGVRVRGAGAQRLQQY<br>PHGQPFSCVPLDAPPGTRAVQLPYALVNDDHCDCADGSDEPGTSACSGAGGYFVCDADVGAPSIHASFV<br>DDGVCDCCDGSDEYAGRTHCQNVCDAQWERAIQESERKVGAFQRALAKRKRMEKDGWTLLAKDRDEI<br>NASAALDTEGSLKKVGEYDAHIEELGRLVKRWEDGLARNPTSDINLTVSESAGQSTSAEADSELTTESDT<br>VQGAETMDVLSKWPPSRCADFISAWPSEKEEKLRGFLPHQLVDSFVSATERLCKIMPFTSCVHQDAERES<br>FHALSIDARLLAAKSCVDAIQQERKLLLDKVQDRQSQAAERIRTLDANHARFPGVRVLRDNCYRSPLGA<br>YEYEICPLVKVLQYEHGRQIAKLGDFKSLMSIESSVRMDFRLGDYCWGRSRRSITVDLACDESEAIVEVSE<br>PSQCKYHMVFSTAAVCEDGMLDVASRQLDTLRSTRHGSQVPASDPRDEL |

**Table S2.** *Cont.*

| Enzyme | ***Porphyridium* sequence** |
|---|---|
| MANIa | MQDASLEHQQQCVAIDFVYSMLSEEAKARFAIERFGLDPFRRVGSYAAYKQQLRNARGCSSTLVSPESSS<br>MHASACAGVPTKAPAPYVFEEHDDIQAKADAIRNATRGAFLAYTFYAFGSDELAPLSRGGVNNFGGMG<br>VTIIDSLSTLYMMDLMEEYALARAWVENQLSFERVGEVVVFETIIRVVGGLASTFQLTGDELFLRKAEEL<br>GKLLGFAFHSPSGVPFPLCHLGRRVCYAKTSHNEMIPIAEAGSIQLEFRALSAMSSDPFIQGIRFSADDFFRL<br>VDSYFEAGEVRVDGNVSMSGLLPSKINFRTGRFKSSMHMLGAPSDSYYEYLFKLWVQSGYTERHLFEKF<br>RNVVRDTIRYLLRRSPALGLYYVFELSGGQPITKMDHFSCFFPATLASACSLPFAPLSNAERAEWMELAE<br>MLAETCHEMYSRSPSGLAPEHVLFDTGKRDWVMFGSYEQRPEAIEAFLFLWRTTRNPKYRDWAWSIFER<br>IQQHSRTAEGAYATLSKARSRRPPKADRMHSFLISETFKYLYLMFQPDHVLPMHLFVLNTEAHPLLLQPH<br>GLLPPQAQDASKDGGTSAGSLP |
| * UGGT | VSVDAPRTWLVSASAAHVDLDNVVLAPQVDKDVEKKRTILAEYVLDAVIISGSAWENAYAPGQDEQEG<br>KGAVAQSVQGLQLALKRFGGQLVSDTVVMQNVGYYQLRATQPSRLRVEMIGAGRDVFVFEATGEPYV<br>GVMLDSXXXXXXXXXXXXXXXXXXXXXXXXXXXXXXXXXXXXXXXXXXXXXXXXXXXXXXXXXXXX<br>XXXXXXXXXXXXXXSAGRAWWQRHVPVHFLTAKSKEPIHVFSVASGHLYERFLRIMMLSASRQSSRPIK<br>FWLLGNYLSPMFKAALPAFAAEHGFDYELVAYQWPPWLRSQSERQRVLWAYKILFLDVLFPLDVSHVIF<br>IDSDQVVRGDLAELLSYQPLPHGAPYAFVPFCDSRVDVEGFRFWKHGYWRGVLGERPYHISALFLVDLR<br>RFRRIAAGDALRVQYQLLSRDPASLSNLDQDLPNSMNKPGGLPIASLPLDWLWCETWCAEDTKWRARTI<br>DLCNNPMTKEPKLVSAKRIIPEWIQLDKEATKSMARILAVNASVSCNVGEKNSAEEP |
| CALNEX | MDDEELARLMEAYGGGGFGGMGENYEDEDPYGGLGAGGFGDMPPPGLELDDLVIDSGVDSEAYSPPDA<br>PASALFLETFQRNAFDEDRWVYSSKAQYNGRFVLGGGRAPGIHGDKGAMLSEKARFYGAVAMLPEPVV<br>VAHGDKLVFQYEVKFDSGLTCSGAYMKLPKSPFATPDVFDNSVQYSIMFGPDKCGDTAKVHVIIQSEHPT<br>TGKLTEHHLTNPPAPFIFGSETHLYTLVLDVAAQTYEVRVDGDVKKAGSLAHDFEPPFQPPSTIPDAKDTK<br>PADWEDEPRIPDPSATKPADWDEDAPLFIPDESATKPDDWLEDEEPQIPDPSAVKPDEWEDSEDGAWQAP<br>LIENPKCVDNGCGPWVAPQIANPDYKGKWTAPMIDNPKYVGPWAPREIENPDYYKVEQVTLLPIAALAF<br>EIWAMDYGIIFDNVYLGTSVEDAEAFANATTVVKRAAETKKSEHTAKKDASDGNSKIKNQVLDAADAV<br>ANALEVVLSPIDALLRKHGLDVYVDAALDFVGSHPLIPSVGIPLVLVVFFLVLTAHRKKQTRSSRTTAVPD<br>VXICEEDGRAASGRCCGACTVSGAIRDHTRAEAGSGGXRRRKAEH |
| CALRET | MSRARVWQCVLRIAAVASACGFCAAGRWDLSKKPENGAVPFYTMPVVEPPAHAYLWEDFQQYKTSFF<br>QVKPGDTEATSWMYARGRGADGAPEIGSIDPLWYRVDKGIGFRKRQRQHYKVARKLDIDTIPDGFTIQF<br>DVRCKAFWTCSGLFWKLLAAPLNSVQDFKDTSPSSIVFGPDRCNEKSRVLVIITTKNPVSGEYEEHVLQN<br>APEPHNHVYKATNLYRLSLFFERGEAVVAVNDKEYVYSLDNDFEPPFQPRKMVDDPADSKPSDWVDER<br>EVVDLDDRQPDDWDETQEPWIPDTSVQKPDDWLEEENAFMKDPNVRKPDFWDDEEDGPWQESWITNP<br>LCLTGKCGTWHQPQIPNPNFRGKWKPRTIPNPDFKGEWQPRKIPNPTYYEIDSVQSIMLPVAGVALDVLV<br>SDYNIWFDNLYVGRSDSEAKYLAEETSKKKQFYETYFEAYPPTVDSEGIPLKEKPWQEMRTHDSQKGAA<br>ETAAKDEL |

* Due to missing information in our genomic data, we have only partial data for the UGGT; the line of X's represent this missing information.

© 2014 by the authors; licensee MDPI, Basel, Switzerland. This article is an open access article distributed under the terms and conditions of the Creative Commons Attribution license (http://creativecommons.org/licenses/by/3.0/).
